# On the Importance of the Sound Emitted by Honey Bee Hives

**DOI:** 10.3390/vetsci7040168

**Published:** 2020-10-31

**Authors:** Alessandro Terenzi, Stefania Cecchi, Susanna Spinsante

**Affiliations:** Dipartimento di Ingegneria dell’Informazione, Universitá Politecnica Delle Marche, 60131 Ancona, Italy; a.terenzi@pm.univpm.it (A.T.); s.spinsante@staff.univpm.it (S.S.)

**Keywords:** bee hive monitoring, real-time monitoring, sound measurement, swarming detection, queen bee detection, sound analysis

## Abstract

Recent years have seen a worsening in the decline of honey bees (*Apis mellifera* L.) colonies. This phenomenon has sparked a great amount of attention regarding the need for intense bee hive monitoring, in order to identify possible causes, and design corresponding countermeasures. Honey bees have a key role in pollination services of both cultivated and spontaneous flora, and the increase in bee mortality could lead to an ecological and economical damage. Despite many smart monitoring systems for honey bees and bee hives, relying on different sensors and measured quantities, have been proposed over the years, the most promising ones are based on sound analysis. Sounds are used by the bees to communicate within the hive, and their analysis can reveal useful information to understand the colony health status and to detect sudden variations, just by using a simple microphone and an acquisition system. The work here presented aims to provide a review of the most interesting approaches proposed over the years for honey bees sound analysis and the type of knowledge about bees that can be extracted from sounds.

## 1. Introduction

**Figure 1:**
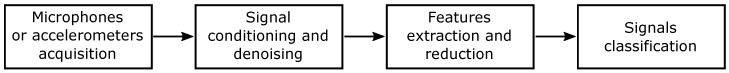
Typical workflow for vibroacoustic signal analysis and classification. First, the sound is acquired inside or outside the hive using microphones or accelerometers. Then, the recorded signal is usually filtered or resampled to remove noise and unwanted frequencies; then, features are extracted from the signal, exploiting different algorithms. If necessary, a features reduction process is applied, based on algorithms such as Principal Component Analysis (PCA). Finally, the data are passed to a classification algorithm to detect the colony health status: typical classifiers are based on Support Vector Machine (SVM), Linear Discriminant Analysis (LDA), and Machine Learning (ML).

Among insects, honey bees are well known for their positive effects on a multitude of different scopes. They do not only produce honey, beeswax, royal jelly, and propolis, but they are at the basis of the plants pollination, playing a key role in the proliferation of both spontaneous and cultivated flora. Recent years have seen an increase in bee mortality, due to several different factors. One of the most recent and dangerous syndromes affecting honey bee colonies is the Colony Collapse Disorder (CCD), which is characterized by a sudden disappearance of honey bees from the hive [1,2,3]. Many bee scientists agree that the decline of honey bee colonies is the result of multiple negative factors working independently, in combination, or synergistically to impact honey bee health [2,4]. Large bee mortality can result in a loss of pollination services with negative ecological and economic [5] impacts that could significantly affect the maintenance of wild plant diversity, wider ecosystem stability and crop production [6,7]. Solutions such as robotic pollination were proposed in recent years, but they were already shown to be technically and economically unacceptable solutions, currently posing substantial ecological and ethical risks [8]. In this context, the necessity of a continuous and intensive monitoring of bee hives’ status emerges clearly, in order to safeguard and protect these important insects. Over the years, several monitoring systems have been proposed in the literature: most of them are based on the measurement of several hive parameters such as temperature, humidity, carbon dioxide, and weight [9,10]. In recent years, a great improvement has come from the integration of web technologies and smart sensors. In [11], a web-based monitoring system built upon sensors and a cloud architecture, to monitor and follow bees’ behavior, is described. In [12,13,14,15], different approaches for heterogeneous wireless sensor networks technologies to gather data unobtrusively from a bee hive have been described. Approaches based on computer vision have been proposed as well: in [16], a system to track bees in a 50 Hz frame rate 2D video of the hive entrance close view is presented. In [17], a real-time imaging system for multiple honey bees tracking and activity monitoring, by counting the honey bees entering and exiting the bee hive, is proposed. Despite all these approaches having shown good results, they still require remarkable computational power, several different types of sensors, and sometimes they impose some modifications to the hive physical structure. A non-invasive technique to monitor the bees’ status relies on sound analysis [18]. Vibration and sound signals are used by bees to communicate within the colony [19,20]. Modalities of vibroacoustic signal production include gross body movements, wing movements, high-frequency muscle contractions without wing movements, and pressing the thorax against the substrates or another bee [21,22,23]. Vibroacoustic signals modulate behaviors that affect swarming, and the queen’s behavior during swarming. In fact, it has been proved that there is a strict correlation between the frequencies of vibroacoustic signals, the amplitudes detected inside the honey bee hives, and the forecasting of events like swarming. The sound can be recorded by means of microphones or accelerometers placed in specific spots inside or outside the hives, and then it can be analyzed to detect the colony health status according to a workflow schematically represented in a general fashion in Figure 1.

This paper aims to provide an up-to-date review on honey bees’ sound analysis approaches, discussing the pros and the cons of each technique, following their historical evolution. The paper is organized as follows: Section 2 provides a review of the scientific literature and how the research on sound emitted by bees evolved along time. Patents related to the analysis of sounds from bees are presented in Section 3, while Section 4 focuses on recent results. Finally, Section 5 provides some concluding remarks and insights for future investigations and developments.

## 2. Literature Review

### 2.1. The Early Works

The topic of analysing the sound emitted by honey bees has always raised the interest of many scientists from different fields. Among the first ones to make some observations on honey bees’ sound was Aristotle who lived in the third century BC. In one of his writings [24], he observed that, when a swarming is imminent, a particular sound is produced continuously by the bees, several days before the swarming event. Later on, one of the early works came from the 17th century AD, in particular one of the first descriptions of queen piping was given by Charles Butler [25], who reported in his book that the bees produce two different sounds. A century later, Francis Huber [26], studying the birth of a new queen, discovered that the first young queens born emit a sound called tooting, and that other young queens, who are still sitting in their cells, respond with another sound called quacking.

### 2.2. The 20th Century and the First Modern Study

During the second half of the XX century, thanks to modern electronics, deeper and more accurate studies on the sound emitted by honey bees were carried out. In 1957, Frings et al. [19] published an article showing that bees react to different sounds. In particular, it was demonstrated that signals of certain frequencies and amplitude produce an almost total cessation of movement of workers bee and drones. Some years later, in 1964, Wenner et al. [27] performed one of the first spectral analyses on the honey bee’s sound. Using a spectrograph and a microphone placed inside the colony, he discovered information about the sound produced during the waggle dance and after the birth of a new queen. The waggle dance is used by bees to communicate within the colony the information about the distance and position of flowers. The work shows that, during the dance, bees emit a specific pulsed sound, in which the number of pulses is proportional to the distance between the colony and the flowers. Wenner also deepened the study about tooting, showing that this sound is composed of two different pulses, the first is a long pulse of 1 s duration followed by shorter pulses, with a fundamental frequency of 500 Hz and some overtones. The quacking has a lower fundamental frequency and starts with shorter pulses. In 1974, Simpson et al. [28] performed a study to better understand the queen bee piping sound [29]. Using a modified speaker attached to a hive, a sinusoidal 650 Hz vibration signal was generated, in order to obtain a signal similar to the one produced by queen bees. When the signal was applied to four hives containing very small colonies with unmated queens, all the four colonies swarmed. The second experiment was carried out with sixteen bigger colonies, each with a young queen bee. This time the sound was applied only to eight colonies which swarmed, leaving a portion of their bees in the hive. The other eight colonies which did not receive the sound didn’t swarm. Despite the simplicity of the generated sound, the experiments show that bees react to sound, even if the limited technology available at the time didn’t allow the authors to generate more complex signals that could also work for colonies with an older queen bee.

In 1985, Dietlein et al. [30] performed a longer study acquiring and analyzing the sound from three different colonies for over a year. The setup used involved two different microphones: one placed inside the hive (for the recording of bees’ sound) and the other one placed between the inner cover and the telescoping outer cover of the hive (to capture the background noise). The microphones were both connected to an analog circuit, which, thanks to a differential amplifier, subtracts the background noise, acquired from the outer microphone, from the honey bees’ sounds. The amplitude of the signal was recorded hourly for a year, showing that there are some significant variations in the amplitude and frequencies of the sound produced in different seasons. Moreover, a frequency analysis has also shown that the spectral content of the signal changes with the seasons and the hour of the day. This study shows an interesting prospective on long-term sound analysis inside the colony, but one of the main drawbacks is probably the analog circuitry used to process the signal, i.e., the analog subtraction between the signals generated by the microphones and the amplification could introduce an additive noise on the signal. One year later, in 1986, Michelsen et al. [20] performed a study more focused on the sound emitted during the waggle dance, analyzing both the vibration and the airborne sound produced. The idea was to better understand which mechanisms are used to communicate during the waggle dance: since it is performed inside the hive in the dark, bees cannot rely on their sight to see the dance, and they must use other communication means, such as vibration, airborne sound, and chemical transmission. Using both a laser vibrometer and microphones placed inside the colony, Michelsen discovered that no vibration is produced during the dance, and that all of the information is passed through the airborne sounds. In particular, as already discovered by Wenner [27], the waggle sound is a pulsed sound, with 20 ms—long pulses at the frequency of 250–300 Hz. It was also discovered that the waggle sound at 2 cm from the bee who is dancing produces a sound wave with a Sound Pressure Level (SPL) of 73 dB. The second part of Michelsen’s work is more focused on the mechanical aspects of the propagation of sound and vibration inside the colony. In this case, the experiment involves the generation of synthetic comb vibration to better understand the freezing phenomena already described in [19]. Again, as already discovered, bees seem to freeze at specific frequencies, and this led Michelsen to speculate that these particular signals are used by bees to quiet the colony, in order to allow a better transmission of important messages. About ten years later, Eren et al. [31] carried out a study in order to better understand and try to emulate the communication between worker bees and the queen bee. From this study, it results that worker bees are not able to produce sound with frequencies higher than 500 Hz, while queen bees are able to produce almost all workers frequencies and many higher ones. Researchers tried to acquire the sound from over 150 queen bees confined in standard queen bee shipment cases, arranged in a matrix form. The acquired sound has been analyzed and then synthesized thanks to a computer. However, the results seem quite inconsistent, exhibiting a weak response from both queen bee and workers to the synthesized sounds that are more complex than the one proposed in [20,27]. This research topic seems quite promising and interesting: the inconsistent results could be mainly due to the experimental setup used for the queen bee sound acquisition. In fact, putting together more than 150 queen bees is not a normal situation, and the sound produced could be very different from the real one generated inside the colony. To achieve better results, it is desirable to acquire the signals in a more natural situation, trying to avoid stressors acting on the bees.

### 2.3. The 21st Century and the Technological Advancement

Within the last twenty years, technological progress has allowed the application of new solutions such as digital signal processing, ML, and low cost smart sensors, enabling new discoveries. In 2005, Ferrari et al. [32] focused their studies on the swarming phenomena. In particular, researchers acquired signals from three hives for over 270 h, observing nine swarming events. Each hive was equipped with an omnidirectional microphone, placed on top of a hive’s looms under the cover, and one humidity and temperature sensor placed inside the hive in between the looms. In the paper, authors do not provide specific motivation for the positioning of the sensors, but we can speculate about the need for keeping the number of microphones used as low as possible-thus adopting a single omnidirectional one-and for monitoring in-the-hive ambient conditions which could as well be correlated to a swarming event. Acquired data were synchronized and then manually labeled in order to identify the swarming events, but authors do not specify which of the three hives was involved in the process. Each event has been analyzed both in frequency and time domain, in order to find any correlation between sound, temperature, and humidity during swarming. Sound spectrograms have shown that there is a quick change in the frequency content during swarming. Before the swarming, most of the energy content of the signal is around 150 Hz, while, during swarming, it jumps to 500 Hz. Moreover, the joint analysis of sound, temperature, and humidity has shown that, during swarming, there is an increase in the sound amplitude, and, meanwhile, temperature and humidity decrease. According to the authors, this behavior could be due to the ventilation produced by bees ready for swarming.

In 2008, Papachristoforou et al. [33] made an interesting discovery, showing that the sound emitted by honey bees could reach high frequencies, even up to 15–16 kHz. The experiment carried out was focused on sound emitted by the bees during a hornet attack. A hornet was artificially introduced inside the hive and the bees’ reaction was recorded. A frequency analysis based on spectrograms revealed that, during a hornet attack, guard bees produce hissing sounds with a fundamental frequency at around 5 kHz, with several high order harmonics which can reach 15 and 16 kHz. The generated sound seems to have a precise structure and this led to thinking that this is a true signal used for communication and not a simple noise produced under stress. Some studies seem to show that honey bees are capable of picking up sound at over 10 kHz with the sub-genual organ, a chordotonal sensor localized in the proximal part of the tibia of each leg [34]. Such a high-frequency structure of the signal could be motivated by the fact that it makes the sound very distinctive from the background noise of the colony, allowing a clear transmission of the alarm message to the whole colony. This study has an important consequence, since it seems to show that the sound produced by the bees reaches frequencies that many other studies seem to ignore completely. In 2011, Eskov et al. [35] proposed a totally different approach, based on statistical sound analysis. The authors considered the recorded signal as a noise with a specific spectral content. As other types of colored noise, the one produced from the bees also has a precise statistical behavior which could be measured. Data from twelve hives were acquired every night from midnight to 3:00 a.m. within the spring and summer periods. Each recording was divided into one second long fragments which could be analyzed by estimating the relative fluctuations of the signal. Each fragment was first normalized and then smoothed using the Procedure of Optimal Linear Smoothing (POLS) [36] with Gaussian kernel, applied to the integrated sequence of original data. Finally, the sequences of ranked amplitudes of relative fluctuations have been estimated giving a statistical indicator of sound behavior. The paper shows that, when bees are going to swarm, the fluctuation changes and this could be used to predict the swarming event several days before it could happen. This work is quite interesting as it proposes an original point of view and could be used for swarming detection. The main drawback is related to the fact that the authors assume that most of the recorded signal is only noise, and this assumption could be a limit in some cases. In 2013, Howard et al. [37] proposed a classification algorithm for queenless colonies, based on the Stockwell Transform (S-Transform) [38] as a features extraction tool. Four colonies of two different species have been monitored, with two normal colonies and two orphaned ones. Data were first analyzed using the S-Transform, which is a time-frequency representation of the signal derived from the wavelet transform with a modification in the phase of the mother wavelet. A qualitative analysis was carried out, comparing the S-Transform, with spectrograms and Fourier transform of the signal. For the classification and representation of the signals, a Self-Organising Maps (SOM) [39] was used. The SOM is trained with two neural networks and allows a clear representation and classification of datasets featuring high dimensionality. Results show that the SOM approach is able to classify the two states with some issues, probably related to the use of two different bee species, or to the classification algorithm. In 2014, Quandour et al. [40] went beyond the simple sound analysis, using the recorded sound to automatically detect if the colony was healthy or infected by the varroa mite. The varroa destructor is one of the most dangerous honey bees parasites and is considered as one of the factors which is contributing to the higher levels of bee losses around the world [41]. The proposed approach was based on the extraction of four statistical indicators from the sound: peak frequency, spectral centroid, bandwidth, and root variance frequency. These four features were then passed to a Principal Component Analysis (PCA) algorithm to reduce the data dimensionality, and finally the data were input to a classifier to detect if the colony was healthy or infected. Two types of classifiers were used, the former based on SVM and the latter on LDA. Both show good performances allowing the discrimination of the two colony statuses. A notable aspect is the reduced computational costs of the proposed algorithm which was implemented on a low-cost single board computer, but, on the other side, the dataset used in the experiments was too limited to give a reliable benchmark on algorithm performances. One year later, Murphy et al. [42] proposed a complete platform for honey bees’ monitoring. In this work, the microphone is only one of the sensors installed within the hive, and the other parameters acquired include CO${}_{2}$, temperature, humidity, acceleration data. Additionally, an infrared camera inside the hive and a thermal camera outside the hive are installed. Sound is used to detect possible swarming events in a very simple way: the system filters and then estimates the envelope of the recorded sound, and, if the amplitude of the signal rises quickly, the system sends a message to the beekeeper. This simple monitoring system could be used for swarming detection on a very simple hardware with also a reduced power consumption, but, due to its simplicity, it could easily generate false positive warnings. In 2017, Ramsey et al. [43] focused their work on the whooping signal, trying to detect this particular signal in different conditions. Vibracoustic signals of three colonies were acquired in different periods of the year and in different geographical locations, with two colonies placed in the UK and one in France. Each colony was equipped with a high precision accelerometer placed in the center of a brood frame. The whooping signal was detected with a two-step process. First, the spectrogram of the recorded signal is matched to the spectrogram of a template pulse, then the ratio of the cross-correlation product and the Euclidean distance among the two is used to find the pulsed signal. Then, in order to discriminate between a whooping signal and non-whooping signal, PCA and LDA are used. The number of whooping signals recorded shows variations which seem related to weather conditions, geographical positions, and time of the day. The whooping sound monitoring could be used as an indicator for hive monitoring; however, the type of sensor used and its precise positioning inside the colony limit this approach, avoiding a possible large scale implementation of the system. In 2018, Kulyukin et al. [44] published an article where they exploit ML techniques to analyze the sound of the hives. In particular, the main goal was to distinguish the honey bee sound, from the background noise and the cricket chirping noise. Four microphones were placed outside the entrance of six Langstroth bee hives. A sound frame of 30 s was recorded every hour, from May to July. Bee hives were placed in different locations, with many different background noises. Data have been manually labeled into three categories: honey bee, cricket, and background noise. With the obtained dataset, several approaches were tested to classify the data; in particular, an ML approach based on a Convolutional Neural Network (CNN) has been compared to traditional classifiers such as Logistic Regression, k-Nearest Neighbors (k-NN), SVM with a linear kernel, one vs. rest classification, and (M4) Random Forests. The results show that, for these types of problems, an ML approach could be used with success, but, since it wasn’t directly applied to the honey bees’ sound, a similar algorithm could be used only as a preprocessing technique to remove unwanted sounds. In the same year, Cejrowski et al. [45] proposed an algorithm to detect the presence of the queen bee based on sound analysis. The system involves a custom brood frame placed inside the colony, with a microphone, and a temperature and humidity sensor. Data have been acquired from a single hive from February 2017 to August 2017, forcing a critical situation for the bees, by removing the queen bee from the hive. Data acquired with the normal colony and the orphaned colony have been analyzed using a Linear Predictive Coding (LPC) for features extraction. This algorithm is based on the source-filter model of a speech signal, i.e., first, the input signal is used to produce a linear model with a number of poles; then, the resulting set of coefficients is used to model the unknown system. Following the LPC coefficients estimation, the t-distributed stochastic neighbor embedding (t-SNE) [46] algorithm has been used to reduce the data dimensionality and, finally, an SVM algorithm was used to classify the results. The algorithm seems to work well with a good ability of detecting changes in the colony sound; however, the data used are limited, with a single colony and a single orphaned event analyzed. Moreover, with the new queen bee, the algorithm seems to need a new training process while a similar approach should be able to detect orphaned and normal colony situations with no need for such a step.

## 3. Patents

Several patents have also been issued over the years. One of the first was presented in 1957 proposing a particular instrument called the Apidictor [47]. The system presented is composed of a microphone, a vu-meter and a series of tunable analog filters. The idea is to adjust the filters to the specific frequency bands at which bees emit specific sounds (i.e., the piping sound) and then to monitor the activity. In 2007, Bromenshenk et al. [48] submitted a patent to exploit the sound emitted by honey bees to monitor air pollution. The main idea is that, when bees are exposed to sub-lethal concentrations of various airborne toxicants, the generated sounds are different and specific for each pollutant. These sounds can be acquired and stored into a database. Then, when a new sound is acquired inside a colony, it is possible to make a comparison with the database, in order to detect pollutants near the colony. For the comparison, first, the spectrograms of various sounds are extracted and then a classification by means of linear discriminant functions is carried on. In 2012, Brundage et al. [49] proposed a system to detect bees productivity based on sound analysis. The idea was to analyze the fundamental harmonic produced by the bees’ flight, which is in the range between 180 Hz to 260 Hz. The presence of a downward frequency shift in the fundamental frequency corresponds to a flying bee launching from locations around the bee hive entrance. By counting the number of frequency shifts, it is possible to estimate the number of bees which have left the hive during a day, and then it is possible to use these data to monitor the hive productivity. One of the most recent patents is from Bencsik et al. [50], who proposed, in 2015, a solution based on previous research on sound emitted by honey bees [43]. The system involves the use of one or more accelerometers, placed at the center of the brood frame. The acquired signal is then transformed into the frequency domain producing a spectrogram. The spectrogram is then processed with PCA to reduce the data dimension. Finally, a linear discriminant function is used to analyze the signal at a reduced computational cost in order to detect events such as swarming or a Varroa mite infection.

## 4. Recent Results

In 2019–2020, several works have been published on bee sound analysis. Nolasco et al. [51] applied ML techniques to analyze honey bees’ sound and detect the queen bee presence. Data from the Nu-Hive project [52] were used, analyzing sound from two different colonies in normal and orphaned situations. Two features’ extraction techniques were used: Mel Frequency Cepstral Coefficients (MFCC) and the Hilbert–Huang Transform (HHT) [53]. MFCC is a widely known technique for signal representation, in which the coefficients are generated starting from the square of signal spectrogram; then, a triangular filterbank is applied to the signal; finally, the Discrete Cosine Transform (DCT) is applied to the logarithmic output of the filterbank. Regarding HHT, the features extraction technique is based on a combination of two algorithms, i.e., the Empirical Mode Decomposition (EMD), which decomposes the signal into a set of basis functions, and the Hilbert Transform (HT) which transforms each basis function into a time-frequency representation of the signal. Using the SVM classifier, a comparison of the performance of both approaches was presented, showing that the best results were achieved with a combination of MFCCs and HHT coefficients. Some experiments with CNNs were carried out as well, using MFCCs as input data and providing good performances. In the mentioned work, authors exploit ML for orphaned colony analysis; however, the most innovative part i.e., the HHT, has not been used in combination with ML, leaving this aspect to a future development. Within the same year, another work was presented involving the queen bee presence detection: Robles-Guerrero et al. [54] acquired sensor data from five hives, and each hive was equipped with a single microphone placed a the center of a brood frame. Data were acquired from March to April, recording 30-s long frames every ten minutes with a Raspberry Pi 2. MFFCs were first computed and then statistical moments for each mel coefficient were calculated involving: mean, standard deviation, variance, skewness, median, and kurtosis. Lasso regularization was then used to reduce again the dimensionality and finally a Logistic Regression algorithm was used for classification. Some experiments were also proposed using Singular Value Decomposition (SVD) and scatter plots to analyze the behavior and separability of the datasets. Among the five colonies, one lost its queen naturally, while, in other two families, the queen bee was removed intentionally. Two experiments were carried out, the former comparing one orphaned colony with the four healthy ones, and the latter removing two queen bees and comparing the data with two normal colonies, and the queenless one. The classifier was able to separate an orphaned colony from the healthy one with a good accuracy. In the same year, another study on sound based swarming detection was published [55]. Data from the open source bee hive project [56] were taken, using MFCCs and LPC as features. As classifiers, two different algorithms were used, the former based on Hidden Markov Model (HMM) and the latter on a Gaussian mixture model (GMM). Several experiments with different combinations of classifiers and features have been carried on, showing that the best solution is MFCCs in combination with an HMM classifier. The work also shows some experiments focusing on the performance degradation due to a high level of background noise in the recorded signals. Again, as in other works, the main drawback is the limited dataset size, since only three swarming events were available and were used for the experiments with only 90 min of recordings for the training phase. Two other studies were published in the same year, which more deeply analyze another point, i.e., how to extract the information from the recorded sound inside the colony. In [57,58], the authors proposed some innovative approaches for the features extraction problem. Many previous research works focused their work on the classifier, using only spectrograms or at least MFCCs to extract important information from the sound. In these two works, some innovative approaches for features extraction are discussed, focusing on orphaned colony situations [57] and swarming [58]. Well known approaches such as spectrograms and MFCCs are compared with innovative algorithms, based on Hilbert–Huang transform, and wavelet transform [59]. Wavelet transform (WT) allows the generation of a time-frequency representation of the signal, and it is based on the decomposition of the signal with specific basis functions called *mother wavelet*. Similar to the Fourier transform, the mother wavelet is translated in time and compressed in amplitude to represent the original signal. Two types of wavelet transforms are available, i.e., the discrete wavelet transform and the continuous one. The results presented seem to show that these approaches are able to better distinguish different signals, and this will be more deeply investigated in a future work where these approaches will be combined with classification algorithms. Another study proposed in 2019 is from [60]. Here, the authors proposed a Raspberry Pi-based acquisition system, able to collect the following parameters: images from the hive entrance, sound, weight, external temperature and humidity, internal and external light level, internal temperature, and air quality. Data are analyzed to detect changes in different periods; weight is also used for swarming detection. A simple analysis of recorded sound is performed by means of a Fourier transform. One of the most recent contributions in this field is from Ramsey et al. [61]. In this work, the authors improve the results of their previous work [43]. In particular, using the same dataset, they propose two different methodologies to predict swarming. One is based on the extraction of one hour logarithmic averaged spectra; then, the cross-correlation product between the spectra and three discriminant functions is calculated. Finally, a linear discriminant function is used to detect swarming. The discriminant functions are estimated by a specific algorithm which analyzes spectrograms of swarming events to estimate the discriminant curves. The second approach is based on a long-term estimation: the algorithm analyzes ten days of vibrational data, acquired from midnight to 5:00 a.m. Ten spectrograms are firstly estimated and then the Fast Fourier Transform (FFT) of each spectrogram is performed on the time course of the magnitude of all the uploaded spectral frequencies for each day, yielding two-dimensional Fourier transform images (2DFT). The FFT is then further calculated over each pixel of the series of 2DFTs found in the preceding days, obtaining a three-dimensional Fourier transform (3DFT). The 3DFT spectrograms are cross correlated with specific discriminant functions and eventually the swarming is predicted using a linear discriminant function. The results seem to show that the first approach is more reliable, providing less false positive swarming events. The second part of the article is focused on the structure of tooting and quacking signals. The results are quite interesting, opening to the possibility of having a reliable swarming prediction. The only problem in the proposed approach is the type of sensor used, which has a high cost and is not suitable for a large scale application. Finally, another recent work is presented in [62], where a combined study is considered by crossing the results of the analysis of the sound generated inside the hive by the honey bees, with other common parameters such as temperature, humidity, CO${}_{2}$ and hive weight, and weather conditions, acquired with several sensors. In this work, the changes in the weight produced during the swarming phenomena are related to the changes in the sound amplitude. Moreover, the other sensors’ variations are analyzed in a normal day and during longer periods, showing trends related to the honey production and the colony activity.

## 5. Future Works and Conclusions

**Figure 2:**
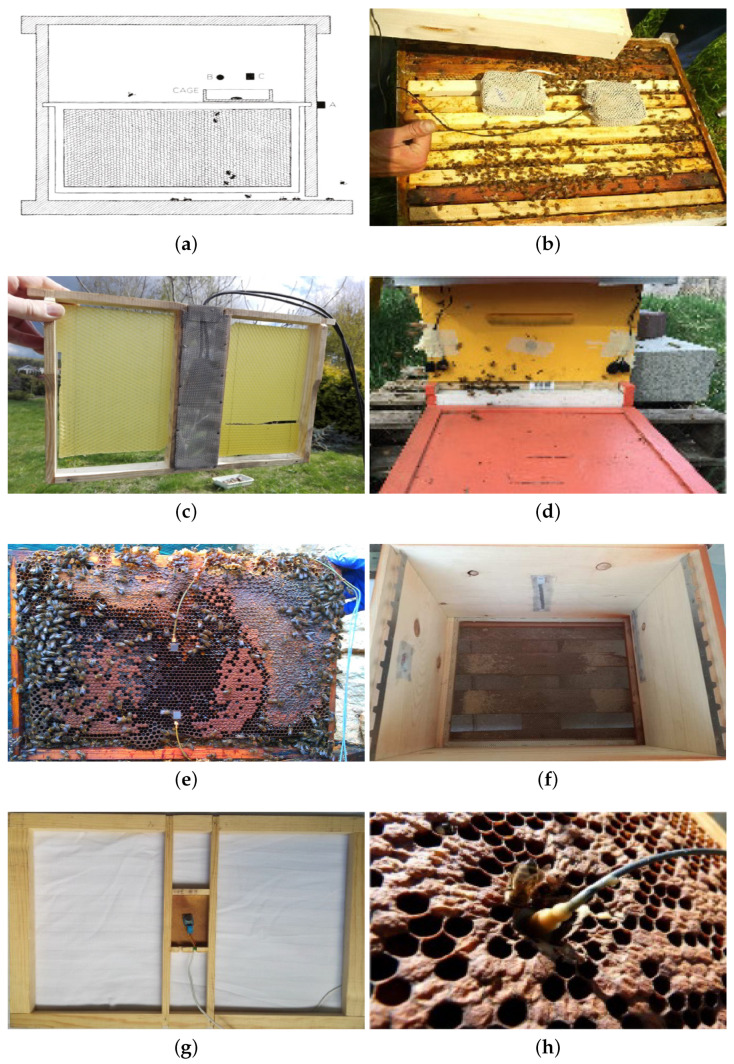
Different microphones and accelerometers placement inside the colonies, based on different approaches. In particular: in (**a**) from [27], B is the microphone used and it is placed above the queen cage. (**b**) shows the microphone placement of [63]: the sensors are placed upon the brood frames inside a cage to protect them from propolization. (**c**) shows the solution adopted in [45], where a custom frame with the sensors inside has been chosen. (**d**) refers to the approach proposed in [44], with microphones placed outside the hive, so that a cage to protect against propolization is not necessary. In (**e**), accelerometers placement proposed in [43] is presented: the sensors exploit vibrations and do not suffer from propolization problems. (**f**) belongs to approaches presented in [51,52,57,58]: the microphones are hidden inside the hive walls, and a grid is used to protect them. (**g**) from [54] shows a custom brood frame. Finally, (**h**) from [61] shows accelerometers positioning similar to the one proposed in [43].

In this paper, following the historical path, a complete overview of the state-of-the-art of approaches and techniques for honey bees sound analysis has been presented: Table 1 shows a summary of all the proposed approaches analyzed in this work, while, in Figure 2, the microphones and accelerometers placement used in several works are visible. Signal analysis techniques, such as HHT, wavelet transform, and multidimensional FFT and LPC have been applied for the first time to the analysis of honey bees’ sound and have been compared to classical approaches based on Fourier transform and MFCCs. Classification algorithms have been discussed as well: linear discriminant approaches have been improved using more sophisticated discriminant functions; other classifiers such as CNNs, SVM, GMM, and HMM have been applied and compared with more traditional approaches. Future works will more deeply analyze these recently proposed approaches, combining new features extraction techniques with innovative classification algorithms.

The issue of collecting datasets of significant dimension and having them available should also be more deeply analyzed: indeed, many proposed works lack dataset dimensionality. Future works should take into consideration this aspect since bigger and labeled datasets could improve significantly the quality, reliability, and significance of the obtained results. Finally, once the best combination between features extraction and classifier has been found, the development of a low-cost system able to monitor the beehive health is a crucial implementation aspect.

## Figures and Tables

**Table 1:** Summary of the state-of-the-art of approaches discussed in this work.

Approach	Description	Applications	References
Spectrograms	The sound is recorded and then analyzed using spectrograms, searching for changes in the harmonic content of the signal.	Analysis of waggle dance, analysis of piping and tooting. Swarming detection. Measuring bees reaction to hornet attack.	[20,27,30,32,33]
Tone based sound synthesis	A loudspeaker or a shaker is placed inside the hive, different tones at different amplitudes and frequencies are generated and bees reaction is monitored.	Find the frequency at which bees react with movement cessation. Reproduce the harmonic generated by the queen bee, to stimulate a swarming.	[19,20,28]
Amplitude monitoring	Amplitude and envelope of the recorded signal is used to detect different behaviors.	Changes of the amplitude in different seasons and conditions. Measuring of SPL during waggle dance. Swarming detection.	[20,30,42]
Bees sound synthesis	The bees sound is firstly recorded and then analyzed and synthesized by means of a computer. The synthesized sound is then reproduced inside the colony and bees reaction is monitored.	Measure the response of worker bees to the synthesized queen bee sound.	[31]
Noise analysis	The recorded sound inside the colony is considered as a noise with a specific statistical behavior. Some statistical indicators are extracted from the sound, changes in the statistical indicators are related to specific colony behaviors.	Swarming detection and prediction.	[35]
Statistical indicator analysis	From the recorded sound, peak frequency, spectral centroid, bandwidth and root variance frequency are extracted. PCA is used to reduce the dimensionality of the indicators and finally SVM or LDA is used to classify the signals.	Detect the presence of Varroa destructor inside the colony.	[40]
Whooping detection	Precision accelerometer inside the colony are used to record the bees vibrations. Spectrograms of vibrations are cross correlated with a pulse signal to detect pulsed signals, LDA and PCA are then used to isolate whooping signals.	Measuring the variation of the whooping signal during different seasons and geographical locations.	[43]
Bees sound detection	The sound is acquired at the hive entrance. Spectrograms of the recorded sound are classified using different algorithms such as, CNNs, logistic regression, SVM, k-NN, one vs. rest and random forest.	Distinguishing the honey bee sound, from the background noise and the cricket chirping noise	[44]
LPC sound analysis	Sound acquired inside the hive is analyzed using LPC as features extraction algorithm. T-SNE algorithm is then used to reduce dimensionality, and finally SVM is used to classify the signals.	Queen bee presence detection.	[45]
HHT and MFCC analysis	Recorded sound inside the colony is analyzed using MFCCs and HHT as features. CNNs and SVM are then applied to classify the signals.	Queen bee presence detection, swarming detection.	[51,57,58]
MFCC analysis	MFCCs are estimated from the recorded signal. Lasso regularization is then used for dimensionality reduction and finally logistic regression algorithm is used for classification.	Queen bee presence detection.	[54]
Wavelet analisys	Wavelet transform is applied to the recorded signal to analyze the sound and detect different behavior.	Queen bee presence detection, swarming detection	[57,58]
MFCC and LPC analysis	MFCC and LPC are used as features, HMM and GMM are used as classifier.	Swarming detection.	[55]
Multimensional FFT	Two and three-dimensional spectrograms are generated starting from the sound recorded using accelerometers placed inside the colony. A discriminant function is then used to classify the signals and detect specific events using two different algorithms.	Swarming detection and swarming prediction.	[61]
